# A Practical Guide to the Mental Capacity Act 2005: Putting the Principles of the Act into Practice

**DOI:** 10.1192/pb.bp.115.052787

**Published:** 2017-02

**Authors:** Martin Curtice

The Mental Capacity Act 2005 (MCA) – which applies specifically to England and Wales – pervades several aspects of daily clinical care within many clinical as well as non-clinical environments, such as care homes.

The authors of *A Practical Guide to the Mental Capacity Act 2005* – whose backgrounds are social work and advocacy – observe that ‘Seven years on […] the MCA is still not being adhered to nor fully embraced within practice’. Their aim was to produce a ‘theory-to-practice breakdown of the MCA’ and this was readily achieved with a fluent and erudite style of writing and continued emphasis on the practical aspects of implementing the MCA. There were useful case studies and checklists for practice, as well as practical top tips such as videotaping advance statements. A minor gripe would be that, if anything, such checklists and top tips could have been used more often throughout the book; for example, at the end of each chapter. The most useful chapter was that on best interests – very salient practical advice was afforded on the best interests process and assessments, including how to chair meetings and using documentation. This chapter tried to demystify the abstract concept of best interests by conceptualising such decisions as complex and less complex. Another strong chapter – probably reflecting the authors' expertise in this area – was on advocacy and empowerment, which examined the various roles of the independent mental capacity advocate within the MCA process.

The undoubted highlight was the evocative account of the 2004 case of *HL v UK* which was the catalyst for the introduction of Deprivation of Liberty Safeguards (DoLS) legislation, to plug the now legally infamous ‘Bournewood gap’. HL was a patient with autism and challenging behaviour who was admitted to hospital on an informal basis. He was regarded as being compliant with care but unable to consent to admission; however, this was found to be a contravention of Article 5 of the European Convention on Human Rights (the right to liberty). The account is written by HL and his carers Mr and Mrs E. Although events regarding HL and his carers began in 1993, the account is a fascinating perspective of one of the most, if not the most, important mental health cases in legal history in terms of its potential impact on tens of thousands of people, carers and clinicians on a daily basis. The authors provide useful views on how and why the DoLS legislation has not been implemented well so far.

Overall, this is an excellent short text which should be required reading for those involved in care touching upon the use of the MCA, and would be ideal for medical and nursing students. But with the Court of Protection seemingly currently engaged in trying to crystallise the core essence of DoLS legislation – and with further recent key judgments emerging in the areas of best interests, end-of-life care and DNACPR (do not attempt cardio-pulmonary resuscitation) – it seems likely that this, as well as other similar guides, will need to be updated again in the near future to keep the readership up to date with key developments.

